# Allopregnanolone, the Neuromodulator Turned Therapeutic Agent: Thank You, Next?

**DOI:** 10.3389/fendo.2020.00236

**Published:** 2020-05-14

**Authors:** Graziano Pinna

**Affiliations:** Department of Psychiatry, The Psychiatric Institute, University of Illinois at Chicago, Chicago, IL, United States

**Keywords:** brexanolone, allopregnanolone (3α,5α-THP), postpartum depression, fast-acting antidepressant, GABA_A_ receptor, 5α-reduced steroids, 5α-reductase, 3α-HSD

## Introduction

Allopregnanolone, today best known as brexanolone and marketed as Zulresso™ for the treatment of postpartum depression is part of only two recently Food and Drug Administration (FDA)-approved fast-acting antidepressants, with esketamine nasal spray, an NMDA receptor antagonist used in treatment-resistant depression being the other (1).

The trajectory, lasting 80 years, that brought allopregnanolone from its discovery (2) in 1938 in the adrenal glands, to understanding its fast non-genomic mechanism in potentiating membrane neurotransmitter receptors, including GABA_A_ receptors (3, 4), underlying its role in acute and chronic stress (5–7), discovering its powerful non-sedative pharmacological effects as anxiolytic and antidepressant agent in animal models and humans (8–10), to the design of the first clinical trials for postpartum depression (11), and finally to the shelves of the clinics in 2019, is regarded as one of the best examples of translational drug development in neuropsychopharmacology (12, 13).

This article redraws the most significant milestones in allopregnanolone discoveries and evaluates future perspective for a new generation of neurosteroid-based treatments in neuropsychiatry. The role of allopregnanolone as a potential biomarker for mood disorders and its pharmacological mechanism in improving behavioral deficits will be discussed.

## Allopregnanolone: 80 Years of Scientific Discoveries

Following its discovery in the adrenal glands (2), Baulieu's laboratory observed (1981) that allopregnanolone can be produced in brain in a manner unrelated to peripheral renovation rates (14). This finding led to coin the term “*neurosteroid*” to define a chemically identical steroid specifically produced by the brain as opposed to “*neuroactive steroids*,” coined by Paul and Purdy (15), which defines steroids produced peripherally that reach and act in the brain. However, it took 25 years to demonstrate that allopregnanolone and its biosynthetic enzymes, 5α-reductase type I (5α-RI) and 3α-hydroxysteroid dehydrogenase (3α-HSD), are expressed in glutamatergic neurons in cortex, hippocampus and basolateral amygdala, and in long-projecting GABAergic neurons in reticulus thalamic nucleus, striatum, central amygdala, and cerebellum but not in glial cells of rodent and human brain (16–20). In 1986, Paul's laboratory observed allopregnanolone is a potent positive allosteric modulator of GABA's action at synaptic and extrasynaptic GABA_A_ receptors (3, 4, 21). Costa and Guidotti' laboratories later cloned and described the function of 18 kDa translocator (TSPO), involved in gating cholesterol entry into the inner mitochondrial membranes, where cholesterol is converted to pregnenolone, the precursors of all neurosteroids (22, 23). Acute stress in rodents fast induces allopregnanolone biosynthesis underlying its role in stress response and demonstrating allopregnanolone present in brain is synthesized independently from peripheral glands (24). However, prolonged stress in rodent models of behavioral dysfunction correlated with downregulated allopregnanolone biosynthesis in corticolimbic circuitry regulating fear responses, anxiety-like, and depression-like phenotypes (7, 25, 26). The first evidence suggesting that allopregnanolone is involved in the etiopathology of depression originated by studies in rodents and depressed patients (27–29). Evidence showed that treatment with the SSRI, fluoxetine normalized the stress—induced decrease of allopregnanolone in rodent brain as well as its lower levels observed in CSF/serum of patients with depression (25, 28, 30). This finding was corroborated by observing SSRIs act as *selective brain steroidogenic stimulants* (SBSSs), increasing selectively allopregnanolone in a manner independent from SSRI mechanisms, underlying a novel mechanism of classical antidepressants (7, 29, 31). Endogenously-produced allopregnanolone in corticolimbic neurons modulates the fine-tuning of GABA_A_ receptors for GABAmimetic, GABA_A_ receptor agonists and positive allosteric modulators. This function underlies allopregnanolone's neurophysiological role. This finding also suggested that allopregnanolone, by this mechanism, may regulate emotional behavior in corticolimbic circuitry (32). Indeed, decreased allopregnanolone biosynthesis in these neurons occurred in association with behavioral dysfunction that are reminiscent of deficits observed in the spectrum of mood disorders (17, 19, 20). Independent laboratories, meanwhile, discovered that allopregnanolone enhances tonic inhibition in δ-containing GABA_A_ receptors, that pregnancy reduces GABA_A_ γ and δ-containing subunits, and that two membrane binding sites on GABA_A_ receptors mediate activation and potentiation of neurosteroid signaling (21, 33–35). Preclinical studies of stress-induced allopregnanolone biosynthesis downregulation contributed to the discovery of several neurosteroidogenic targets through which, agents that increase allopregnanolone biosynthesis, are beneficial in improving behavioral deficits (36–39).

**Figure 1:**
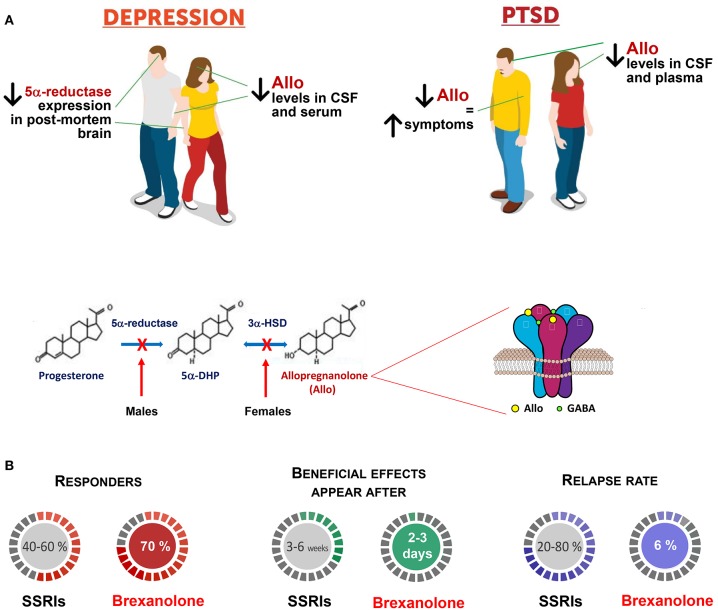
Brexanolone is superior to traditional antidepressants in the treatment of mood disorders. **(A)** Patients with mood disorders, including major unipolar depression and PTSD, exhibit serum, plasma, CSF, and brain reduction of allopregnanolone levels and/or biosynthesis, which includes the enzymes, 5α-reductase type I (5α-RI), and 3α-hydroxysteroid dehydrogenase (3α-HSD) [(18, 28, 30); reviewed in (10, 42)]. In women with PTSD, progesterone, and the immediate allopregnanolone precursor, 5α-dihydroprogesterone (5α-DHP) levels fail to change and their ratios with allopregnanolone and pregnanolone (allopregnanolone's equipotent GABAergic isomer), concentration in the CSF and plasma points to a possible deficit at the 3α-HSD enzyme expression/function levels (43). Likewise, in PTSD male patients, the CSF allopregnanolone concentrations are decreased for an apparent deficit in 5α-RI expression/function, which negatively correlates with PTSD and depression symptoms (43–45). Thus, the concentration and the ratio of allopregnanolone with its parental neuroactive steroids can suggest deficits in their enzymatic pathway, which may unveil biomarkers of sex hormone-related mood disorders. Allopregnanolone's mechanism of action includes activation of mainly extrasynaptically-expressed GABA_A_ receptors. GABA_A_ receptor offers two residues for neurosteroid action; one is located between α and β subunits, and the second is a cavity on α subunits (34). The efficacy of neurosteroids at GABA_A_ receptors is greatly enhanced by the α*βδ*-containing GABA_A_ receptor subtype, which is characteristic of tonic inhibition mediated by extrasynaptic receptors (21). Allopregnanolone plays a pivotal neurophysiological role by modulating the fine-tuning and strength of GABA_A_ receptors (32). By this mechanism, allopregnanolone appears to regulate emotional behavior and the pharmacological response of GABA_A_ receptor. Altered GABA_A_ receptor subunit composition has been observed in several pathophysiological conditions, including across the menstrual cycle, changes in hormonal shape during pregnancy, as well as during protracted stress (46). Stress, specifically, results in a GABA_A_ receptor composition with increased sensitivity for neurosteroids and neurosteroid-like molecules (e.g., synthetic allopregnanolone analogs) [(29); reviewed in (42)]. These observations are in support of treatments that stimulate allopregnanolone biosynthesis for the therapeutic management of stress-induced psychiatric disorders, for which traditional anxiolytics or antidepressants are ineffective. **(B)** Brexanolone, a β-cyclodextrin-based parenterally administered soluble formulation of allopregnanolone is marketed as Zulresso™ and it is the first and only specific treatment for postpartum depression. Brexanolone is one of only two recently FDA-approved fast-acting antidepressants. In clinical trials, women with postpartum depression treated with brexanolone improved their symptoms compared with placebo in 2.5 days. Symptoms were measured before and after treatment. Follow-up studies showed that women receiving the treatment maintained the therapeutic gains for at least 30 days (41). Side effects include risk of sedation or loss of consciousness during treatment. For these reasons women who undergo treatment will be monitored by a healthcare professional in a healthcare setting. Other side effects may include sleepiness, dry mouth, flushing of the skin or face. A clinical trial using the orally-active allopregnanolone analog, SAGE 217 has recently failed for non-compliance issues that were noted with about 10% of patients presenting no blood drug levels. However, statistical significance was achieved at days 3, 8, 12, and 15 in patients with measurable drug concentration levels of SAGE-217. Hence, these allopregnanolone derivatives are highly promising in the treatment of mood disorders, from postpartum depression to major depression and, probably, in PTSD, which, as mentioned above, is characterized by low allopregnanolone levels (43, 45). Another approach is to use neurosteroidogenic drugs (38). These agents may selectively elevate allopregnanolone levels by stimulating enzyme activity/expression levels where a deficit emerges thereby improving mood symptoms avoiding a global expression of allopregnanolone levels.

Collectively, these and many more observations in the field by many talented neurosteroid scientists, led to clinical trials that demonstrated the efficacy of intravenous allopregnanolone in postpartum depression (40, 41). Given the remarkable pharmacological efficacy of this novel therapeutic, on March 19th, 2019, the FDA approved intravenous allopregnanolone (i.e., brexanolone) as the first specific treatment for postpartum depression (Figure 1). Clinical studies are currently evaluating the pharmacological efficacy of an orally-active allopregnanolone called SAGE 217 for the treatment of major depressive disorders (47). A new era of fast-acting, short-course, long-lasting, neurosteroid-based treatments is born.

## Mechanisms Linking Biosynthesis of Allopregnanolone to Mood Disorders

Allopregnanolone, a positive allosteric modulator of GABA's action at GABA_A_ receptors (3, 4, 15, 48), is deficient in mood disorders (28, 30, 43, 45). Allopregnanolone and progesterone change significantly in pregnancy and after parturition (33, 49). The increase in plasma progesterone throughout pregnancy triggers upregulation of allopregnanolone levels, which reaches the highest blood concentrations during the third trimester (49, 50). Following childbirth, these neurohormones abruptly decrease (51, 52). Among the hypotheses linking allopregnanolone decrease and post-partum depression, the suggestion that allopregnanolone drops quicker and to lower levels than in mothers who fail to develop post-partum depression is particularly intriguing. This effect may be resulting from abnormal neurosteroid enzyme expression. Mechanistically, GABA_A_ receptor function may fail to adapt to the rapid allopregnanolone level decline during the weeks following parturition (53). Studies conducted in estrous cycle in rats demonstrated that the drastic decrease of progesterone concentrations during diestrus is associated with overexpression of extrasynaptic α4β1δ-containing GABA_A_ receptors in periaqueductal gray, which mediates anxiolytic and mood regulating effects of allopregnanolone in this estrous phase (54, 55). The expression of specific subunits of the GABA_A_ receptor is coordinated with fluctuations in neurosteroid concentrations during menstrual/estrous cycle, pregnancy, and perinatally function (21, 33, 56). Pharmacological treatments, including finasteride and oral contraceptives, that inhibit 5α-RI, which results in a blood and brain allopregnanolone decrease also affect subunit expression of GABA_A_ receptor and are associated with mood symptoms and suicide and are part of postfinasteride syndrome (57, 58). Post-finasteride syndrome, in addition to depression, anxiety and cognitive deficits also induces sexually-related side effects, such as loss of libido, erectile dysfunction, decreased arousal and difficulty in achieving an orgasm that persist despite drug withdrawal (58). Evidence suggests during pregnancy and across the estrous cycle a switch of extrasynaptic δ with synaptic γ2 subunits may be operative (33, 56). Rapid and dynamic changes among synaptic and extrasynaptic GABA_A_ receptor conformation in areas that regulate cognitive functions and emotions, including the hippocampus have been reported (59).

Altogether, stressful condition, hormonal changes, pharmacological treatment (e.g., finasteride, oral contraceptives) may coordinately change GABA_A_ receptor expression resulting in alterations in receptor function underlying mood disorders. They may alter GABA_A_ receptor pharmacology in response to anxiolytics (42). Conversely, allopregnanolone, its analogs, and neurosteroidogenic agents may offer a therapeutic advantage for disorders that arise by these deficits.

## Allopregnanolone-Based Treatments

To contrast the rapid post-partum depletion of allopregnanolone and the rise of mood deficits, directly supplementing synthetic neuroactive steroids or their analogs, may offer a quick strategy in treating post-partum depression and other mood disorders linked with the drastic drop in endogenous allopregnanolone (53, 60). Following this concept, brexanolone, a β-cyclodextrin-based parenterally-administered soluble formulation of allopregnanolone, was developed and FDA-approved for treating post-partum depression. In an open-label study, a single brexanolone IV administration showed rapid and long-lasting antidepressant effects in severe post-partum depression (40). Safety and efficacy was further confirmed in two double-blind, randomized clinical trials (41). Brexanolone presumably acts by reinstating normal allopregnanolone levels, and thereby tuning GABAergic neurotransmission function, promptly improved symptom severity in with post-partum depression patients (Figure 1). However, it still remains to be clarified the precise treatment targets, including levels of endogenous allopregnanolone, verify altered biosynthetic enzyme expression/function, and GABA_A_ receptor assembly modifications pre, during, and post-brexanolone treatment. The elevation of brain derived neurotropic factor (BDNF) is also conceivable among allopregnanolone's mechanisms (61). Each of these factors may be critical for understanding why—and for whom—brexanolone is best indicated to improve mood symptoms. First, deficient allopregnanolone levels may be critical for predicting who may benefit from varying doses of direct neurosteroid replacement (via brexanolone or other allopregnanolone analogs). Second, baseline allopregnanolone concentrations are crucial to select the most effective brexanolone dose and avoid unwanted side-effects, including excessive sedation (62). Third, by directly affecting both the HPA and HPG axes, allopregnanolone may alter expression of key biosynthetic enzymes (e.g., 5α-RI and 3α-HSD) involved in neurosteroid synthesis. Indeed, the HPA axis is modulated by GABAergic neuron activation within the hypothalamus (63). Allopregnanolone potently inhibits HPA axis activity and repress stress elevation of ACTH and corticosterone (64, 65). This finding suggests that allopregnanolone administration may alter HPA axis responsiveness by affecting gonadal steroid concentrations (e.g., estradiol) with documented roles in maintaining expression/function of neurosteroidogenic enzymes (e.g., 3α-HSD) and sustainably change endogenous neurosteroids production (66).

Collectively, these reports suggest that more studies are needed to verify the diverse mechanisms involved in brexanolone treatment.

## What's Next? Allopregnanolone as a Biomarker for Mood Disorders

While converging evidence suggests a neurosteroid biosynthesis deficit involvement in the underlying neurobiology of mood disorders, the yet unanswered question is whether allopregnanolone biosynthesis (allopregnanolone levels and expression of rate-limiting biosynthetic enzymes) provide a reliable biomarker to prevent mood disorder, predict occurrence, diagnose, and indicate treatment selection. Another valid option suggests analyzing neurosteroid biosynthesis relative to GABA_A_ receptor subunit dynamic changes. GABA_A_ receptor expression and neurosteroid biosynthesis in post-partum depression and in general in mood disorders remains underinvestigated. Furthermore, analysis of neurosteroids that positively modulate GABA_A_ receptors (allopregnanolone and pregnanolone), and of their sulfates (e.g., pregnanolone sulfate), that inhibit NMDA-mediated tonic neurotransmission, which results in neuroprotection and cognitive improvement (67), has been poorly investigated. Establishing predictive biomarkers of treatment response will enable follow-up analysis of neuroactive steroid biosynthesis and GABA_A_ receptor composition that will help predict whether brexanolone pharmacological effects are associated with permanent neurobiological improvements or, alternatively, whether GABAergic functional deficits may anticipate relapses following drug discontinuation. Assessing a *biomarker axis*, indicating the dynamic changes of several inter-related neurobiological deficits will facilitate a more thorough diagnosis of mood disorders as well as predict which patients will likely respond to treatment. This will increase efficacy and limit occurrence of side-effects.

In neuropsychopharmacology establishing reliable biomarkers and efficient treatments is urgently needed. Currently, patients show large non-response and relapse-rate to traditional antidepressants and significant side-effects.

## Conclusions

Eighty years of neurosteroid research originated from many talented neuroscientists around the world guided investigations that from the discovery of allopregnanolone led to its approval as a fast-acting agent to treat post-partum depression. One of the most significant achievements still remaining to be accomplished in neuropsychopharmacology and, in general in psychiatry, is the assessment of valid biomarkers to predict, diagnose, select, and treat patients more efficiently, avoiding drug non–responders and side-effects. Neurosteroidogenic targets have been recently suggested that may result in new drug development (38, 68). The opportunity of increasing allopregnanolone levels and improving deficits with functional foods (69) is an emerging novel approach to treat mood disorders in a more natural way without exposing pregnant women to drugs.

## Author Contributions

The author confirms being the sole contributor of this work and has approved it for publication.

## Conflict of Interest

The author declares that the research was conducted in the absence of any commercial or financial relationships that could be construed as a potential conflict of interest.
