# Corrigendum: Allopregnanolone, the Neuromodulator Turned Therapeutic Agent: Thank You, Next?

**DOI:** 10.3389/fendo.2020.00507

**Published:** 2020-07-30

**Authors:** Graziano Pinna

**Affiliations:** Department of Psychiatry, The Psychiatric Institute, University of Illinois at Chicago, Chicago, IL, United States

**Keywords:** brexanolone, allopregnanolone (3α, 5α-THP), postpartum depression, fast-acting antidepressant, GABA_A_ receptor, 5α-reduced steroids, 5α-reductase, 3α-HSD

In the original article, there was an error. “Allopregnanolone, today best known as brexanolone and marketed as Zulresso™ for the treatment of postpartum depression is part of only two recently Food and Drug Administration (FDA)-approved fast-acting antidepressants, with esketamine nasal spray, an NMDA receptor antagonist used in treatment-resistant depression with suicidality being the other.” **However, SPRAVATO**^®^ **(esketamine) CIII Nasal Spray was approved by the FDA last year to treat treatment-resistant depression in adults when used along with an oral antidepressant, and is not indicated for the treatment of suicidality**.

A correction has been made to the **Introduction** section, First paragraph:

“Allopregnanolone, today best known as brexanolone and marketed as Zulresso™ for the treatment of postpartum depression is part of only two recently Food and Drug Administration (FDA)-approved fast-acting antidepressants, with esketamine nasal spray, an NMDA receptor antagonist used in treatment-resistant depression being the other (1).”

The authors apologize for this error and state that this does not change the scientific conclusions of the article in any way. The original article has been updated.

